# Assessing the utility of metabarcoding for diet analyses of the omnivorous wild pig (*Sus scrofa*)

**DOI:** 10.1002/ece3.3638

**Published:** 2017-11-26

**Authors:** Michael S. Robeson, Kamil Khanipov, George Golovko, Samantha M. Wisely, Michael D. White, Michael Bodenchuck, Timothy J. Smyser, Yuriy Fofanov, Noah Fierer, Antoinette J. Piaggio

**Affiliations:** ^1^ Fish and Wildlife Conservation Biology Colorado State University Fort Collins CO USA; ^2^ USDA, Wildlife Services National Wildlife Research Center Wildlife Genetics Lab Fort Collins CO USA; ^3^ Department of Pharmacology The University of Texas Medical Branch Galveston TX USA; ^4^ Department of Wildlife Ecology and Conservation USA5 USDA, Wildlife Services University of Florida Gainesville FL USA; ^5^ Tejon Ranch Conservancy Frazier Park CA USA; ^6^ USDA, APHIS, Wildlife Services San Antonio TX USA; ^7^ Department of Ecology and Evolutionary Biology Cooperative Institute for Research in Environmental Sciences University of Colorado Boulder CO USA; ^8^ Present address: Department of Biomedical Informatics College of Medicine University of Arkansas for Medical Sciences Little Rock AR USA

**Keywords:** blocking primer, CO1, diet, feral swine, metabarcoding, trnL

## Abstract

Wild pigs (*Sus scrofa*) are an invasive species descended from both domestic swine and Eurasian wild boar that was introduced to North America during the early 1500s. Wild pigs have since become the most abundant free‐ranging exotic ungulate in the United States. Large and ever‐increasing populations of wild pigs negatively impact agriculture, sport hunting, and native ecosystems with costs estimated to exceed $1.5 billion/year within the United States. Wild pigs are recognized as generalist feeders, able to exploit a broad array of locally available food resources, yet their feeding behaviors remain poorly understood as partially digested material is often unidentifiable through traditional stomach content analyses. To overcome the limitation of stomach content analyses, we developed a DNA sequencing‐based protocol to describe the plant and animal diet composition of wild pigs. Additionally, we developed and evaluated blocking primers to reduce the amplification and sequencing of host DNA, thus providing greater returns of sequences from diet items. We demonstrate that the use of blocking primers produces significantly more sequencing reads per sample from diet items, which increases the robustness of ascertaining animal diet composition with molecular tools. Further, we show that the overall plant and animal diet composition is significantly different between the three areas sampled, demonstrating this approach is suitable for describing differences in diet composition among the locations.

## 1 INTRODUCTION

Obtaining detailed diet information for many animal species is difficult due to both the arduous effort required to directly observe and physically identify food items from stomach contents (Pompanon et al., 2012; Schley & Roper, 2003). Traditional stomach content analyses are often limited to the detection of diet items that have been recently consumed, as many food items are rapidly digested or quickly become indiscernible and, are thus underestimated using these traditional techniques (Ballari & García, 2014; Schley & Roper, 2003; Valentini, Pompanon, & Taberlet, 2009). Woody plants, on the other hand, are often difficult to digest, and animals are known to simply chew the roots in order to extract the sap and starches, only to later expel the tough woody tissue (Wood & Roark, 1980). This limits the amount of discernable material remaining for the visual assessment of diet composition through direct observation (Wood & Roark, 1980). DNA‐based tools can be used to infer diet composition as the DNA for many indiscernible ingested items such as eggs, animals, and plants is often still present (Schley & Roper, 2003; Valentini et al., 2009). High‐throughput sequencing (HTS) allows for the parallel sequencing of target amplicons across many samples and makes the comparative analyses of diets from multiple fecal samples increasingly tractable, particularly for fauna with complex behaviors (van Doormaal, Ohashi, Koike, & Kaji, 2015; Marini, Franzetti, Calabrese, Cappellini, & Focardi, 2009; Podgórski et al., 2013) or omnivorous feeding habits (De Barba et al., 2014). These features combined with the ever‐increasing size of DNA sequence reference databases improve the ability to detect rare or seasonal food items that might otherwise be missed (De Barba et al., 2014; Valentini et al., 2009). Recently, HTS approaches, specifically DNA metabarcoding with various markers, have been applied to obtain deeper insight into the diet of several species of megafauna, such as the American bison (*Bison bison*) (Bergmann, Craine, Robeson, & Fierer, 2015), gazelles (*Gazella dorcas*) (Ait Baamrane et al., 2012), other large African herbivores (Kartzinel et al., 2015), and omnivorous brown bears (De Barba et al., 2014).

An accurate description of dietary breadth and feeding behaviors is imperative for understanding the ecological impacts of invasive species, especially those with variable food preferences, such as the omnivorous and invasive wild pig (*Sus scrofa*; hereafter wild pigs) (Ballari & García, 2014). From the early 1500s onward, wild pigs were introduced to North America multiple times by Europeans either as deliberate introductions for the establishment of game populations or as an incidental consequence of free‐range livestock practices (Seward, VerCauteren, Witmer, & Engeman, 2004). In the late 1800s, Eurasian wild boar was also introduced into the continental United States for big game hunting (Rollins, 1993; Seward et al., 2004). Newly introduced Eurasian wild boar interbred with the previously established free‐living domestic pigs, creating an array of hybrids that exhibit a wide range of phenotypic variation and life history traits (Bevins, Pedersen, Lutman, Gidlewski, & Deliberto, 2014; Goedbloed et al., 2013; McCann, Malek, & Newman, 2014). Wild pigs have become the most abundant free‐ranging exotic ungulate in the United States (Seward et al., 2004) and, among big game, are second only to white‐tailed deer (*Odocoileus virginianus*) in the number of individuals harvested by hunters (Kaufman, Bowers, & Bowers, 2004; Mayer & Brisbin, 2009) . These large populations of wild pigs are ecologically destructive (Barrios‐Garcia & Ballari, 2012; Bevins et al., 2014) and are responsible for spreading invasive plants (Bankovich, Boughton, Boughton, Avery, & Wisely, 2016; Boughton & Boughton, 2014) and pathogens (Cooper, Scott, de la Garza, Deck, & Cathey, 2010; Ruiz‐Fons, 2015). These issues have contributed to the estimated $1.5 billion in damages and control costs each year within the United States with similar levels of economic losses in other nations (Bevins et al., 2014; Choquenot, Lukins, & Curran, 1997; Pimental, 2007).

Wild pigs are omnivores, yet traditional diet analyses have shown that they primarily consume plant material (Ballari & García, 2014; Barrios‐Garcia & Ballari, 2012). However, the monogastric digestive system of pigs is not as efficient in breaking down cellulose, hemicellulose, and structural carbohydrates as the polygastric digestive system common among other ungulate species (Ditchkoff & Mayer, 2009). Thus, wild pigs prefer easily digestible plant material high in protein, starch, and simple sugars such as acorns and other mast resources (Ditchkoff & Mayer, 2009). When preferred food resources become scarce, wild pigs will switch to other locally abundant and easily digestible resources such as fungi, ground‐nesting birds (and their eggs) (Rollins & Carroll, 2001a, 2001b), amphibians, reptiles, small fossorial mammals (Wilcox & Van Vuren, 2009). Wild pig feeding behavior can introduce negative impacts on native wildlife populations (e.g., predation of deer fawns) or species of special concern by federal and state wildlife management agencies (Beach, 1993; Seward et al., 2004). In some cases, wild pigs will prey upon livestock (e.g., newborn goats) (Beach, 1993; Pavlov & Hone, 1982; Seward et al., 2004) or consume agriculturally available food items like corn and peanuts (Ballari & García, 2014; Barrios‐Garcia & Ballari, 2012; Ditchkoff & Mayer, 2009) or livestock feed and mineral supplements intended for livestock (Cooper et al., 2010).

Given the known biases and challenges of stomach content analysis, and the need to identify impacts of wild pigs to plants and wildlife populations, we developed and evaluated a method to use HTS to increase knowledge of the dietary breadth of this ecologically and economically destructive invasive species (Ballari & García, 2014; Pompanon et al., 2012; Schley & Roper, 2003; Valentini et al., 2009). Our goal was to test the feasibility of determining both plant and animal diet composition of wild pigs through PCR amplification and sequencing the trnL (UAA) intron and the cytochrome c oxidase subunit 1 (CO1) marker gene regions from wild pig fecal samples. Primers sets for both the CO1 and trnL (UAA) marker genes are available, or can be constructed, to amplify short fragments of DNA that can be recovered from feces or gut contents of many animals (Deagle, 2006; Symondson, 2002; Zaidi, Jaal, Hawkes, Hemingway, & Symondson, 1999). The trnL (UAA) intron is highly conserved throughout the plant kingdom and has been used for the molecular detection of food crops and allergens (James & Schmidt, 2004). Thus, the use of the trnL (UAA) intron for plant identification and systematics has been well established (Taberlet et al., 2007). Similarly, CO1 is a mitochondrial‐encoded marker which has been used widely in animal systematics (Chen, Giles, Payton, & Greenstone, 2000; Symondson, 2002). The Barcode of Life Data System uses CO1 as one of the primary marker sequences for animals, due to its effectiveness in delineating the majority of animal assemblages (Ratnasingham & Hebert, 2007). The only caveat in using CO1 for diet analysis within wild pigs, or any vertebrate host, is the co‐amplification of host DNA along with diet. Host DNA template is more abundant and less degraded than DNA from diet items (Deagle, Eveson, & Jarman, 2006; Nejstgaard et al., 2008; Vestheim & Jarman, 2008), which can bias or restrict the molecular detection of food items (Green & Minz, 2005; Polz & Cavanaugh, 1998). Given these challenges, we also investigated the utility of blocking primers to limit the co‐amplification and sequencing of the host CO1 DNA (Vestheim & Jarman, 2008). The current study included wild pig fecal samples from three states within the United States: Florida, Texas, and California. These areas are known to support high densities of wild pigs (McClure et al., 2015; Snow, Jarzyna, & VerCauteren, 2017), encompass different plant and animal communities colonized by wild pigs, and were selected to represent a broad sample of the diversity of diet items potentially consumed by wild pigs. Demonstration of the differences in diet composition among the three study areas would provide validation that an HTS metabarcoding approach, at a minimum, can resolve course scale differences in diet composition expected between disparate ecosystems.

## 2 MATERIALS AND METHODS

### 2.1 Sample collection

We collected fecal material as either fresh scat collected from transects within 24 hr of defecation following the methodology of Kierepka et al. (2016) (California; 19 individuals) or from fecal material taken directly from the colon of culled individuals (Texas and Florida; 14 and 15 individuals, respectively). California samples were collected from 31 July 2014 through 3 September 2014 and immediately placed on ice in the field, then frozen. In Florida and Texas, a 10‐cm section of colon was removed from freshly euthanized animals and placed on ice in the field and then frozen within the same day. Florida specimens were collected from 13 May 2014 through 28 May 2014, and the Texas specimens were collected from 6 May 2014 through 11 June 2014. For geographical locations, see Table S2. For all individuals, subsamples of the frozen specimens were submitted to the University of Texas Medical Branch (Yuriy Fofanov) and the University of Colorado (Noah Fierer) for DNA metabarcoding.

### 2.2 Metazoan diet analyses (CO1)

Previously published PCR primers used for the amplification of the mitochondrial‐encoded cytochrome oxidase subunit I (COI) were downloaded for evaluation from the Bold Systems Database (Ratnasingham & Hebert, 2007). In‐silico performance was evaluated using CLC Genomic Workbench Primer Identification tool. The primers were matched against the CO1 reference database (July 2014) from Bold Systems focusing on a list of species of interest inhabiting the immediate area of sample collection (Table S1). Due to the degraded nature of fecal‐derived sequences, short CO1 amplicons were preferred (Deagle et al., 2006) (Symondson, 2002) (Zaidi et al., 1999). This process resulted in several potential CO1 primer pairs, which were subsequently tested experimentally in the laboratory. The following primer pair MICOlintF (5′‐GGWACWGGWTGAACWGTWTAYCCYCC‐3′) (Leray et al., 2013) and PolyShortCoiR (5′‐CCNCCTCCNGCWGGRTCRAARAA‐3′) (Carr, Hardy, Brown, Macdonald, & Hebert, 2011) resulted in amplicons of ~200–300 bases and were considered universally optimal for the target taxa of interest (Table S1).

Genomic DNA from fecal swabs was extracted using the MoBio PowerFecal Isolation Kit (Carlsbad, CA) per the manufacturer's protocol. Each PCR was made using the Q5 Master Mix, with 3.5 μl of DNA, 6.2 μl of H_2_O, for a total reaction volume of 12.5 μl. The thermocycling program used an initial step at 95°C for 3 min, a final extension at 72°C for 5 min, and the following steps cycled 35 times: 30 s at 95°C, 30 s at 55°C, and 30 s at 72°C.

Amplicon DNA yields from each PCR were then quantified using Nanodrop 2000 (Thermo Fisher Scientific Inc.) and Quanticus Fluorometer (Promega). All PCRs were normalized to equimolar concentrations and pooled together before purification using the MoBio UltraClean PCR Clean‐Up protocol. Sequencing libraries for each sample were generated in accordance with the Illumina 16S rRNA metagenomic sequencing library preparation protocol. Sequencing was performed on an Illumina MiSeq at the University of Texas Medical Branch Bioinformatics and Genomics Laboratory. Single 501 bp forward reads were generated for the sequencing run. Each individual pig was sequenced twice, once with COI blocking primer and once without.

### 2.3 Pig COI blocking primer

The initial sequencing analysis of *S. scrofa* fecal samples using the universal amplification primers resulted in high relative abundance of host CO1 amplicons and only a limited number of sequences from diet items. To decrease the relative abundance of the host sequences, a blocking primer was developed to limit the amplification of *S. scrofa* CO1 sequences. Due to the lack of specificity of the *S. scrofa* CO1 near the amplification site, dual priming oligomers (DPO) were developed using the approach of Vestheim and Jarman (2008) to block host sequence amplification while minimizing blocking interference with other metazoan sequences. The DPO overlapped with the 3′ end of the forward universal primer extending into *S. scrofa*‐specific sequence and was modified with a C3 spacer at the 3′ end, which produced the following blocking sequence: 5′‐ACCCACCTTTAGCTGGAAACTTAGCCCATGCAGGAGCTTCAGTTGATCTAACAAIIIICTCCCTACACCTT‐C3‐3′. The blocking primer sequence was rigorously tested against metazoan taxa within the BOLD Systems Database and found to be specific to the host. The efficacy of the blocking primer was tested in vitro and was verified to bind to the extracted DNA using both a 10:1 and 1:1 ratio of blocking primers to amplification primers.

### 2.4 Sequence processing

Raw demultiplexed forward and reverse read fastq files were generated via QIIME v1.9.1 (Caporaso et al., 2010) using split_libraries_fastq.py script with quality filtering disabled by setting the following parameters: q 0, max_bad_run_length 250, and min_per_read_length_fraction 0.001. Cutadapt (Martin, 2011) was used to trim the primers from the reads in paired‐end mode. If the primers were not detected (up to 10% mismatch allowed) within the reads, then that read/read‐pair was discarded. For paired‐end data, reads were merged via the fastq_mergepairs command in USEARCH (Edgar, 2010). Sequence denoising, quality filtering (maxee setting of 0.5), PHiX and chimera removal, and OTU (Operational Taxonomic Unit) clustering were implemented via the UNOISE (v2) pipeline (Edgar, 2016b). Taxonomy was assigned via the SINTAX approach (described below) implemented in USEARCH (Edgar, 2010, 2016a). As the primers and blocking primer were optimized for the detection metazoan taxa, any OTUs that were not classified to a metazoan family and contained less than eight reads were discarded prior to all downstream analyses. General analyses and generation of figures were performed in R (R Core Team, 2017) using the following packages: vegan (Dixon, 2009), ggplot2 (Wickham, 2009), reshape (Wickham, 2007), phyloseq (McMurdie & Holmes, 2013), and mctoolsr (Leff, 2016 2016). All individual wild pigs were sequenced once with and without the CO1 blocking primer. R (R Core Team, 2017) was used to compare output of the two HTS run conditions via paired *t* tests and OTU rarefaction accumulation curves (*specaccum* via the vegan (Dixon, 2009) package) to ascertain if we could obtain greater sequencing depth of host diet with the use of the blocking primer.

### 2.5 Plant diet analyses (trnL)

Genomic DNA from fecal swabs was extracted using the MoBio PowerSoil‐htp 96‐well Isolation Kit (Carlsbad, CA). A portion of the chloroplast trnL intron was PCR amplified using the g (5′‐GGGCAATCCTGAGCCAA‐3′) and h (5′‐CCATTGAGTCTCTGCACCTATC‐3′) primers for the trnL gene (Taberlet et al., 2007), but modified to include appropriate barcodes and adapter sequences for Illumina multiplexed sequencing. Unique per sample 12‐bp error‐correcting barcodes were used, as described in Caporaso et al. (2012). Each PCR was mixed per the Promega PCR Master Mix specifications (Madison, WI), with 2 μl of gDNA template for a reaction volume of 25 μl. The thermocycling program used an initial step at 94°C for 2 min, a final extension at 72°C for 2 min and the following steps cycled 35 times: 2 min at 94°C, 1 min at 55°C, and 30 s at 72°C.

Amplicon DNA yields from each PCR were then quantified using PicoGreen fluorometry (Thermo Fisher Scientific Inc.). All PCRs were normalized to equimolar concentrations and pooled together before purification using the MoBio UltraClean PCR Clean‐Up protocol. Sequencing was performed on a single Illumina MiSeq lane with 2 × 150 cycles at the University of Colorado Next‐Generation Sequencing Facility. We sequenced single sample per individual pig. Sequence processing was performed as described above.

### 2.6 Reference databases

FASTA records containing only the trnL amplicon region from Streptophyta and representative outgroup taxa, along with the COI amplicon region from metazoa and fungi, were downloaded via Entrez Direct command‐line tools from GenBank (Benson, Karsch‐Mizrachi, Lipman, Ostell, & Wheeler, 2005; Kans, [Link]). The SINTAX protocol of USEARH (Edgar, 2010) (Edgar, 2016a) was used to create reference databases that correspond to the specific amplicon regions of the trnL and CO1 marker sequences from all downloaded GenBank (Benson et al., 2005) records. PyCogent (Knight et al., 2007) was used to extract the full taxonomic lineage using the gi‐to‐taxid mapping files provided by GenBank. All extracted amplicon regions were dereplicated to 100% sequence identity, and any identical sequence across lineages was collapsed to the lowest‐common‐ancestor (e.g., if several genera contain identical sequence across the amplicon region of interest, the reference taxonomy was set to the family level) using the standard operating procedures suggested by the UTAX and SINTAX protocol of USEARCH (Edgar, 2010, 2016a).

## 3 RESULTS

### 3.1 Host‐blocking primers for CO1

**Figure 1:**
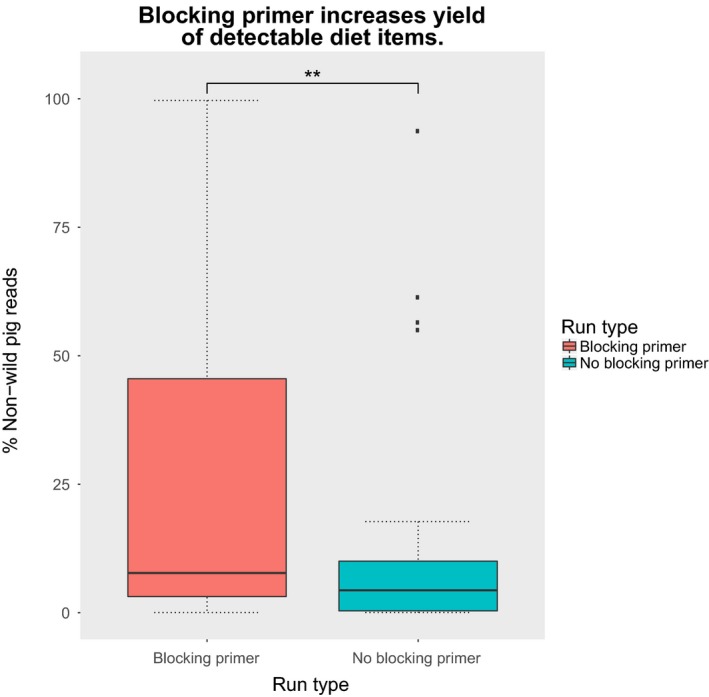
Box‐whisker plot showing a significant (paired *t* test, *N* = 27 per run type, *p*‐value < .01) increase in the percentage of nonhost DNA amplified when using blocking primers versus not using blocking primers

**Figure 2:**
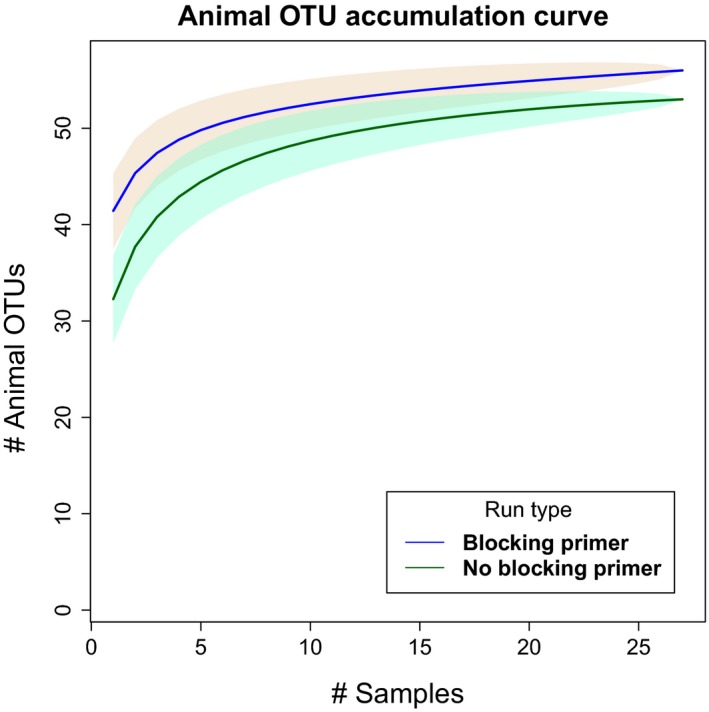
CO1 blocking primer versus nonblocking primer metazoan OTU rarefaction curves across all samples. Deeper access to diet OTUs after bioinformatics QA/QC and host DNA removal

**Figure 3:**
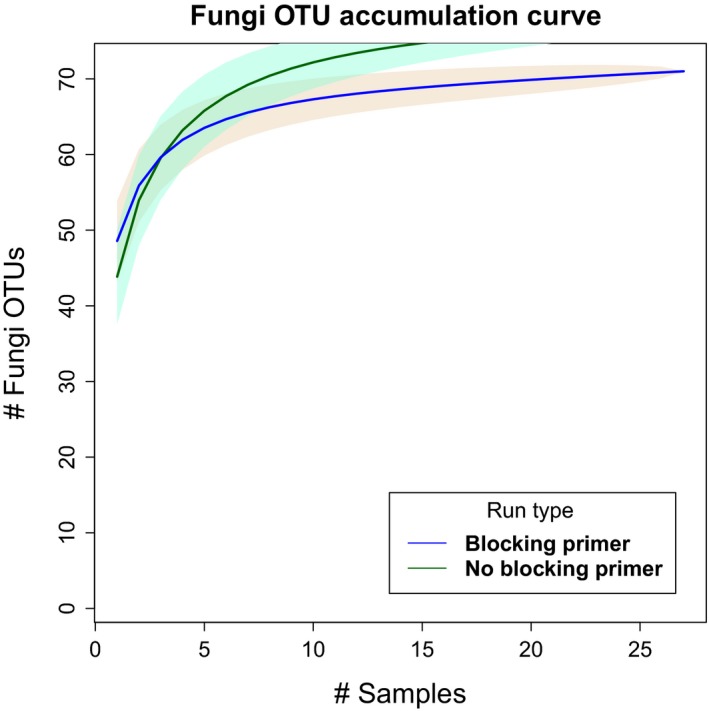
CO1 blocking primer versus nonblocking primer fungal OTU rarefaction curves across all samples. The blocking primer noticeably inhibits fungal amplification

The CO1 host‐blocking primer significantly (*p*‐value < .01) increased the number of metazoan diet sequences recovered by an average of 17% more, compared to runs without blocking primers, postbioinformatic QA/QC and removal of host amplified DNA (Figures 1 and 2). Based on these results, we opted to focus comparisons between landscapes and the interpretation of our results on samples in which the blocking primer was applied as they yielded more OTUs. Additionally, many of the nonblocking primer treatment samples either returned limited sequence data or completely failed to sequence, making these data limited for diet assessment. Although fungi were amplified and sequenced with the CO1 primers, the blocking primer notably inhibited the assessment of fungal diet items (Figure 3) and was excluded from the analysis.

### 3.2 Metazoan diet (CO1)

**Figure 4:**
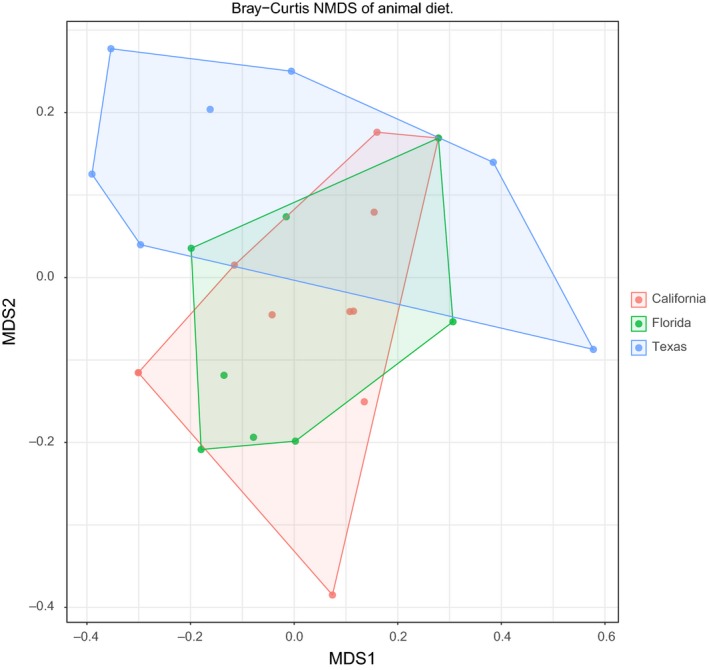
Bray–Curtis NMDS plot based on rarefied metazoan OTUs rarefied to 742 reads per sample (retaining 26 of 43 samples). Pairwise permutational ANOVA revealed that all sites significantly different after correcting by False Discovery Rate (*p* < .05). The percent variation explained at the state level was 13.9%

A total of 270,418 forward reads (truncated to 250 bp) comprising 91 metazoan OTUs across 70 samples were retained for diet analysis upon successful sequencing and post‐QA/QC and host sequence removal. The 43 blocking primer samples represented 15, 14, and 14 samples from California, Florida, and Texas, respectively. Whereas the 27 nonblocking primer treatment samples were comprised of 10, 11, and 6 samples, from the same regions, respectively. Differences in regional diet were confirmed via a Bray–Curtis NMDS plot (Figure 4), produced by rarefying each sample to 742 reads per sample, to balance sequencing depth with the number of samples. All regions were significantly different from one another based on pairwise permutational ANOVA with multiple comparisons corrected for using False Discovery Rate (*p*‐value < .05).

**Figure 5:**
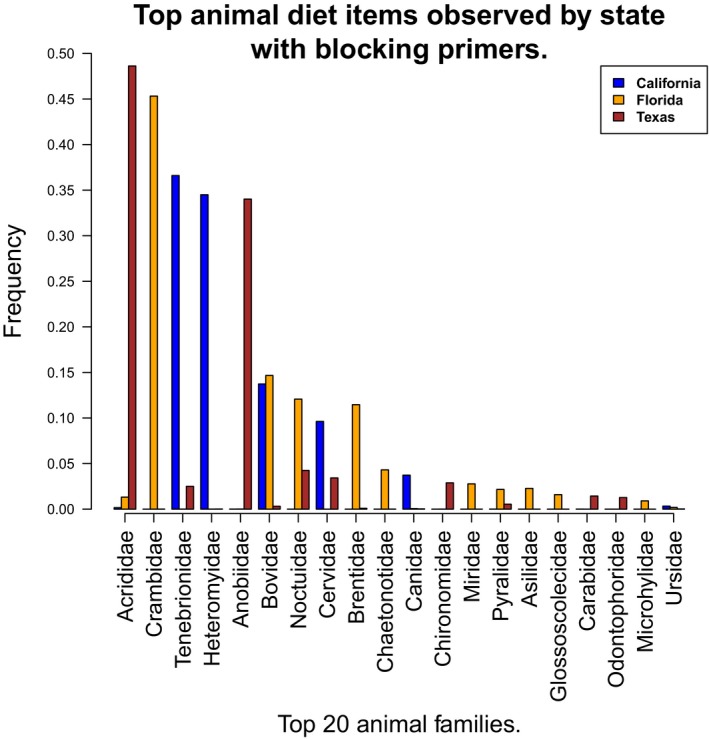
Top metazoan Families by state with blocking primers

**Figure 6:**
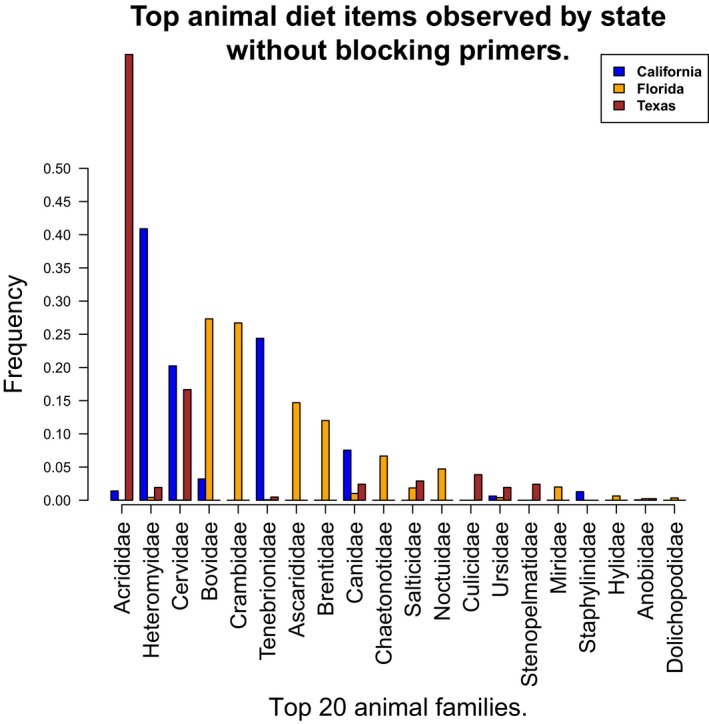
Top metazoan Families by state without blocking primers

Differences in regional metazoan diet can be observed at the family level (Figure 5). California wild pigs had a large portion of their diet consisting of Tenebrionidae (beetles) which commonly live under the bark of oak trees (Fagaceae). Wild pigs in Texas had more classifiable insects within the Acrididae (grasshoppers) and Anobiidae (a family of beetles including wood borers). Finally, Florida wild pigs had abundant Crambidae (moths). There were mammals and birds in the diet from all regions. Notably, we detected quail (Odontophoridae: *Colinus virgianus*) in the diets of wild pigs from Texas, elk (likely Rocky Mountain elk; *Cervus elaphus canadensis*) from California, deer (Cervidae: *Odocoileus* spp.) from Texas, kangaroo rats (Heteromyidae: *Dipodomys* spp.) and deer mice (*Peromyscus* spp.) in CA, the eastern narrow‐mouthed toad (*Gastrophryne carolinensis*) from Florida, and Bovidae (cattle) in all three states. We also observed minor differences in dominant animal taxa between samples with and without the use of the blocking primer (Figures 5 and 6).

### 3.3 Plant diet (trnL)

**Figure 7:**
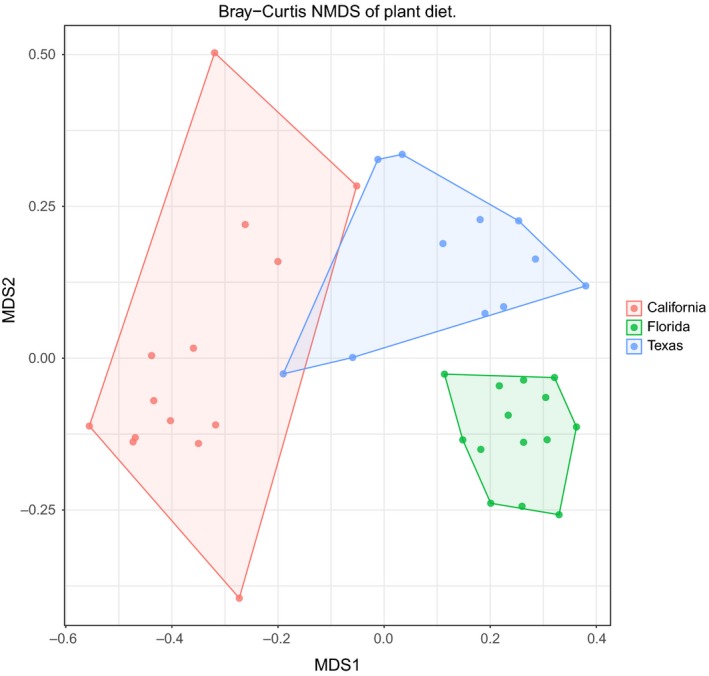
Bray–Curtis NMDS plot based on plant OTUs rarefied to 6,094 reads per sample (retaining 39 of 39 samples). Pairwise permutational ANOVA revealed that all sites significantly different after correcting by False Discovery Rate (*p* < .01). The percent variation explained at the state level was 27.9%

A total of 802,155 merged paired‐end reads, averaging 52 bp in length and comprising 2,480 OTUs (99% similarity) across 39 samples, were retained for plant diet analysis postbioinformatics QA/QC sequence removal. These remaining 39 samples consisted of 14, 14, and 11 samples from California, Florida, and Texas, respectively. The differences in regional diet are exemplified by the Bray–Curtis NMDS plot (Figure 7) which was produced by rarefying each sample to 5,994 reads per sample. All regions were significantly different from one another based on pairwise permutational ANOVA and corrected for using False Discovery Rate (*p*‐value < .01).

**Figure 8:**
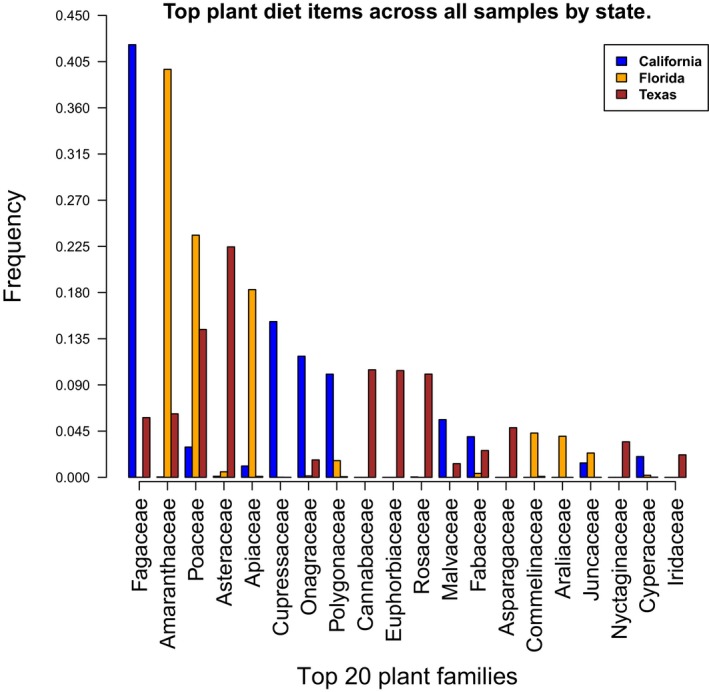
Top plant Families by state

Wild pig samples in California exhibited large amounts of Fagaceae (beeches and oaks) in their diet profiles (Figure 8). This was followed by Cupressaceae (cypress, juniper, redwood), Onagraceae (willow herb/evening primrose family), and Polygonaceae (knotweed/smartweed, buckwheat family). Wild pigs in Florida had large amounts of Amaranthaceae (annuals, leafy vegetables, ornamental plants), Poaceae (grasses), and Apiaceae (celery, carrot, parsley) in their diets. We also detected Carolina redroot (*Lachnanthes caroliniana*) in Florida, a plant often observed in greater abundance after rooting by wild pigs (Boughton & Boughton, 2014). The diets of wild pigs from Texas were dominated by Asteraceae (asters, daisies, sunflowers), Poaceae, Cannabaceae (Cannabis, hops, hackberries), Euphorbiaceae (spurge family), and Rosaceae (many from the genus *Prunus* (edible fruits), roses).

## 4 DISCUSSION

Our results corroborate the benefits of DNA metabarcoding in elucidating the dietary profiles of megafauna as demonstrated previously with other taxa (Ait Baamrane et al., 2012; Bergmann et al., 2015; De Barba et al., 2014; Kartzinel et al., 2015). Despite the benefits of these HTS technologies for diet analyses, there are some technical issues to consider when targeting specific marker genes for diet analysis. When a marker gene of interest is co‐amplified from the host target species, two problems arise: (1) the dominance of host DNA template within a sample can saturate the system restricting molecular detection of diet items and biasing the results, and (2) DNA from diet items are often far more degraded than that of the host, making the detection of such items increasingly difficult to detect (Deagle et al., 2006; Nejstgaard et al., 2008; Vestheim & Jarman, 2008). We found that our initial sequencing attempts primarily returned wild pig sequences, which provided shallow sequencing depth for the characterization of diet items (Figures 1 and 2). This would undoubtedly create challenges for the detection of rare diet items. Based on this outcome, we developed and validated primers that blocked the amplification of pig DNA (Vestheim & Jarman, 2008) and resequenced the samples.

The use of host‐blocking primers provided a significantly (*p* < .01) deeper sequencing for animal diet composition of wild pigs (Figure 1). Further, the introduction of a blocking primer increased the effectiveness of using metabarcoding by increasing the number of diet items detected (Figure 2), as has been shown in other studies (De Barba et al., 2014; Lundberg, Yourstone, Mieczkowski, Jones, & Dangl, 2013; Vestheim & Jarman, 2008). We emphasize that when the objective is the detection of uncommon food items, particularly for invasive species with highly variable diets, it is imperative to use an approach that limits the amplification and sequencing of the host. However, it is difficult to confirm if the blocking primers may have biased the compositional profile of animal diet items as has been reported previously (Piñol, Mir, Gomez‐Polo, & Agustí, 2015). For example, several of the top metazoan families differed between the blocking and nonblocking primer treatment (Figures 5 and 6). It is unclear whether the apparent bias affects our assessment of animal diet composition, as the comparison of blocking and nonblocking treatments is conflated by the significantly lower sampling depth of diet items in the nonblocking treatment (Figure 1). Additionally, the differential abundance of sampled taxa without the blocking primer may reflect stochastic or biased sampling of diet due to host background DNA interference as mentioned above. Thus, researchers should consider the effects of potential blocking primer biases (Piñol et al., 2015) as they would take into account other primer biases (Deagle, Jarman, Coissac, Pompanon, & Taberlet, 2014). However, the animal diet items of greatest interest to natural resource managers (i.e., game species and species of conservation concern) were detected in higher frequency when the blocking primer was applied. The blocking primer does have a noticeable impact on reducing the detection of fungi (Figure 3) which is not surprising as our protocol was optimized for the detection metazoan taxa. If a study demands an understanding of host consumption of fungi, then a more appropriate marker gene such as the internal transcribed spacer (Blaalid et al., 2013; Schoch et al., 2012) should be used.

Variation in food availability and supplementary feeding is often reflected by differences in the geographical locations of wild pig populations (Schley & Roper, 2003). We found significant differences in regional plant diet composition among the three regions we sampled (*p*‐value < .01). Although we also detected significant differences in animal diet composition between these regions (*p*‐value < .05), there was greater variability and thus overlap of animal diet between the sampling locations compared to that of plants (Figures 4 and 7). This pattern likely reflects the opportunistic feeding behavior of individual wild pigs on animals, carrion, feces, and nests (Ditchkoff & Mayer, 2009). Some of this variation may have resulted from differences in sampling, that is, unlike the colon samples from Texas and Florida, California was sampled from fresh scat and are potentially not independent samples (from the same individual sampled at different times).

Wild pigs are known to consume energy‐rich plant food such as acorns, beechnuts, chestnuts, pine seeds, cereal grains, and fruits. (Ditchkoff & Mayer, 2009; Schley & Roper, 2003). This pattern was most clearly observed within the California wild pigs, where oaks (Fagaceae) comprised upward of 40% of the plant diet (Figure 8). However, the California samples were collected from July through August, prior to the peak ripening of acorns in this part of California. The vegetative cover of oaks can be very high in parts of the California study site, and it is possible pigs incidentally ingested oak tissue (e.g., leaves or roots) while foraging for other prey items, or consumed squirrel acorn caches (Ditchkoff & Mayer, 2009). Furthermore, the high preponderance of Tenebrionidae taxa observed within the California samples is not surprising as they are often found in association with oaks (Steiner, 2014). As the California scat samples were collected noninvasively from the landscape, it is possible that a portion of other less abundant insect sequences may have come from larvae that were deposited directly into the scat (Albuquerque & Zurek, 2014).

Interestingly, the diet of a single pig from California almost entirely consisted of sequences mapped to the genus *Dipodomys*, and more specifically to *Dipodomys panamintinus* (Panamint kangaroo rat) and secondarily confirmed via BLASTn (99%–100% identity). The next closest BLASTn hit was to *D. heermanni* at 95%. *D. panamintinus* has been observed at the sampling location (M. White, personal observation); however, the amount of existing sequence data for the *Dipodomys* genus is limited. This intriguing result requires further investigation. Another small rodent, *Peromyscus eremicus* (cactus mouse), was also detected in a single California pig. These results corroborate previous descriptions of wild pigs eating small mammals (Ditchkoff & Mayer, 2009) such as ground squirrels and other fossorial and semifossorial vertebrates (Ditchkoff & Mayer, 2009; Loggins, Wilcox, & Van Vuren, 2002; Wilcox & Van Vuren, 2009). Many of these small mammals are regionally endemic or considered species of special conservation concern by federal or state wildlife management agencies. For example, five taxa of *Dipodomys* are listed in the International Union for the Conservation of Nature Red List (IUCN ‐ Red List), six *Dipodomys* taxa (such as *D. ingens*) are federally listed as Endangered in California, and *D. elator* is listed as threatened in Texas. Given the ability of wild pigs to prey upon a variety of small mammals, this invasive species can be considered another potential risk factor for small mammal populations of special concern where they co‐occur.

Northern bobwhite quail (*Colinus virginianus*) is a popular game animal for recreational hunting. The exponential increase in wild pig populations in Texas over the past 30 years (Bevins et al., 2014) has coincided with the decline of *C. virginianus*. The direct role of pigs in *C. virginianus* declines is difficult to confirm through traditional stomach analysis as they likely target eggs (De Barba et al., 2014; Schley & Roper, 2003; Wood & Roark, 1980), yet we detected a high number of *C. virginianus* sequences within the diet of a single wild pig sampled in Northern Texas. Nest depredation may negatively impact quail recruitment and concomitant hunting opportunities, and decreasing populations of quail have been observed elsewhere where wild pigs are present (Brennan & Kuvlesky, 2005; Rollins & Carroll, 2001a, 2001b). Similar concerns exist for other ground‐nesting game birds such as wild turkey (*Meleagris gallopavo*) (Bankovich et al., 2016; Wood & Lynn, 1977; Yarrow & Kroll, 1989). Given our small sample size, these results suggest a targeted study of wild pigs during quail nesting season could be valuable for understanding their impact on this species and other ground‐nesting bird populations.

We also detected deer and elk (*Odocoileus* & *Cervus*) within the diet of wild pigs in Texas and California, two important game species in these states. This supports prior observations of wild pigs either actively preying upon or scavenging deer and livestock carrion, (as reviewed in Ditchkoff and Mayer (2009)). Active predation, scavenging, or consumption of fecal matter cannot be differentiated with the molecular approach outlined here. Only direct field observation can be used to confirm which occurred. When food supplementation is used to attract deer, invasive wild pigs often compete for these resources and destroy feeding dispensers, displacing deer from the area (Cooper, 2005; Tolleson, Pinchak, Rollins, & Hunt, 1995). Additionally, a survey conducted by Wood and Lynn (1977) showed that 47% of foresters, wildlife biologists, and land managers believed that wild pigs were direct competitors to deer, turkeys (*M. gallopavo*), and small mammals like squirrels (*Sciurus* spp.). These observations were subsequently corroborated, in part, by Yarrow and Kroll (1989), in which they observed seasonal competition between deer and wild pigs for mast and forage, especially during drought when alternate or supplemental food is unavailable. These examples highlight the complexity of wild pig management and the challenges of balancing the control of wild pigs to reduce competition with native game species with the interests of some members of the hunting community that view wild pigs as a valuable game species (Bevins et al., 2014).

The degree by which pant monocultures can be established through the foraging and rooting behaviors of wild pigs may be dependent upon the region and local densities of wild pigs (Boughton & Boughton, 2014; Bueno & Jiménez, 2014). The disturbance caused by rooting can facilitate the growth of plants that are both toxic and unpalatable to cattle (Bankovich et al., 2016; Boughton & Boughton, 2014) but preferred or tolerated by wild pigs. The increase in toxic and unpalatable plants devalues range land by decreasing forge, resulting in reduced herd sizes, which can have a negative economic impact for ranchers (Bankovich et al., 2016). Here, we report the detection of Carolina redroot (*L. caroliniana*) and plants from within the genus Spermacoce (comprising several species of False Buttonweed) from several Florida individuals. Additionally, we also detected coinwort (*Centella asiatica*), in several Florida pigs, which is also known to be associated with low‐forage quality land for cattle grazing (Boughton, Quintana‐Ascencio, & Bohlen, 2010). The promotion of such unpalatable plants on rangeland is economically detrimental to cattle ranchers (Bankovich et al., 2016; Boughton & Boughton, 2014).

Additionally, increasing the level of unpalatable plant species within native Florida grassland pastures has unknown consequences for other popular game species such as northern bobwhite quail, wild turkey, and white‐tailed deer (Bankovich et al., 2016). These species depend upon diverse grassland communities for both forage and cover. Ever‐decreasing plant diversity may result in a habitat that can neither sustain locally threatened species nor continue to provide recreational hunting opportunities. The negative ecological consequences of wild pigs may outweigh the short‐term economic benefit associated with recreational wild pig hunting as it has been shown that it is difficult for recreational hunting to control wild pig densities to a level that imposes minimal impacts on wildlife populations (Bankovich et al., 2016; Seward et al., 2004).

We have shown that not only is the dietary monitoring of wild pigs possible using HTS tools, but can significantly supplement direct observational assessment of property, crop, and rangeland damage by wild pigs. The HTS approach as outlined here and elsewhere (Ait Baamrane et al., 2012; Bergmann et al., 2015; De Barba et al., 2014; Kartzinel et al., 2015; Pompanon et al., 2012) make it tenable and cost‐effective for the public to work with local government agencies to submit fecal samples of culled wild pigs for diet and other analyses. The local experience of ranchers, farmers, and wildlife biologists can be used to supplement and refine HTS tools and reference databases to enhance existing management practices. Finally, molecular metabarcoding reference databases are continually being updated, which will provide greater depth and breadth of taxonomic identification for a variety of marker genes. As new voucher species are added to sequence databases, HTS diet survey data can be continually reanalyzed to classify DNA sequences that may have been previously tagged as “unresolved” or “unclassified” (e.g., classified only to family level) due to the lack of closely related marker gene sequences at the time of a given survey.

Finally, molecular tools should complement, not replace, traditional observational assessment of wild pig feeding behaviors. For example, Wilcox and Van Vuren (2009) developed criteria for identifying vertebrate carrion within wild pig gut contents, by ascertaining the odor, dehydration level, and maggot content of the tissue. Similarly, DNA tools also cannot differentiate between items actively consumed by pigs, versus by‐catch through rooting behaviors (e.g., animals or fungi living in and on plants), or animals and fungi that may have colonized scat after it was deposited.

## 5 CONCLUSION

Wild pigs consume a wide variety of plant, and animal resources present within their invaded range, including species of conservation concern and game species. Spatio‐temporal sampling of feral swine populations should be a major component of future studies, as radical shifts in diet (e.g., large acorn mast events or depredation of nests) can alter management and damage mitigation strategies. Knowing the temporal feeding patterns for various habitats will enable managers to predict when and where wild pigs will travel and can facilitate preventative rather than reactionary management practices (Wood & Roark, 1980). Further, this method will be an effective tool for gaining a more detailed understanding of this invasive species’ impacts to crops, game species, livestock, and other plant and animal species of conservation concern.

## ACKNOWLEDGMENTS AND AUTHOR CONTRIBUTIONS

MSR performed the research, analyzed the data, and wrote the manuscript. AP designed the research and contributed to the writing of the manuscript. KK, GG, and YF designed CO1 blocking primers, sequenced the CO1 amplicons, and contributed to writing of the manuscript. NF sequenced the trnL amplicons and contributed to the writing of the manuscript. SMW, MW, and MB collected the samples and contributed to writing of the manuscript. TS contributed to writing the manuscript. Funding was provided by the USDA National Wildlife Research Center.

## CONFLICT OF INTEREST

None declared.

## DATA ACCESSIBILITY

Sequence data have been uploaded to the Genbank SRA under BioProject PRJNA415437.
